# Concern about addiction is associated with lower quality of life in patients with osteoarthritis: an exploratory, real-world data analysis

**DOI:** 10.1007/s11136-021-02907-0

**Published:** 2021-07-05

**Authors:** Louis P. Garrison, Patricia Schepman, Andrew G. Bushmakin, Rebecca L. Robinson, Leslie Tive, Jerry Hall, Mendwas Dzingina, James Jackson, Mia Berry, Joseph C. Cappelleri, Stuart Silverman

**Affiliations:** 1 Department of Pharmacy, The Comparative Health Outcomes, Policy, and Economics (CHOICE) Institute, University of Washington, Health Sciences Building, H375, 1959 NE Pacific St, Box 357630, Seattle, WA, 98195-7630 USA; 2 Pfizer Inc, New York, NY USA; 3 Eli Lilly and Company, Indianapolis, IN USA; 4 Adelphi Real World, Bollington, UK; 5 Rheumatology Division of the Department of Medicine, Cedars-Sinai Medical Center, Los Angeles, CA USA

**Keywords:** Opioids, Addiction, Quality of life, Osteoarthritis

## Abstract

**Purpose:**

To evaluate the relationship between self-reported concerns about becoming addicted to a medication and health-related quality of life (HRQoL) in patients with osteoarthritis (OA).

**Methods:**

This real-world study used patient-level cross-sectional survey data collected from the US Adelphi Disease Specific Programme (DSP). The DSP for OA selected 153 physicians who collected de-identified data on their next nine adult patients with OA. Each patient completed a disease-relevant survey, which included the Likert-scale question, “I am concerned about becoming addicted to my medicine,” (CAA) with responses ranging from “completely disagree” [1] to “completely agree” [5]. HRQoL was measured by the EQ-5D-5L index value and the EQ Visual Analogue Scale (VAS). A set of ordinary least squares regressions using HRQoL measures as outcomes and CAA as a continuous predictor were estimated. Standardized effect size (ES) was used to gauge the magnitude of effects.

**Results:**

A total of 866 patients with OA completed the survey (female, 61.2%; White, 77.7%; mean age, 64.2 years). Of the 775 patients who completed the CAA question, almost one-third responded that they “agree” (18%) or “completely agree” (11%), while 27% responded “completely disagree” and 20% “disagree.” Regression analyses found that patients who have concerns about medication addiction have significantly different EQ-5D-5L index values and EQ VAS scores compared with patients who do not have this concern (*p* < 0.0001).

**Conclusion:**

Our findings suggest that concern about medication addiction in patients with OA may have an impact on patient HRQoL, with more concerned patients reporting poorer HRQoL outcomes.

## Introduction

Osteoarthritis (OA) is a leading cause of pain and disability among older adults, and is estimated to affect over 27 million individuals in the United States, with further increases in prevalence expected due to an aging population and rising obesity rates [1–4]. OA joint pain, and the related functional limitations and reduced quality of life, account for substantial socioeconomic burden. Total aggregate healthcare expenditures for OA have been estimated at $185.5 billion annually in the United States and are expected to rise [5].

Effective treatment for the symptoms of OA is limited, but recent clinical guidelines recommend a multimodal approach to treat OA optimally, combining physical therapies with pharmacological interventions, such as acetaminophen, nonsteroidal anti-inflammatory drugs (NSAIDs), weak opioids, and other medicines [6–8]. The Osteoarthritis Research Society International (OARSI) recently issued guidance strongly recommending against opioid use for OA-related pain [9], largely over concerns related to opioid addiction or dependency, and the most recent guidelines from the American College of Rheumatology/Arthritis Foundation conditionally recommended their use only after other options had been exhausted [8]. Although controversial, opioids continue to be prescribed for the treatment of pain associated with OA, especially as pain intensity increases [10, 11].

In a previous US treatment preference study of a hypothetical, disease-modifying, pharmacological treatment for OA, patients with OA were willing to accept some degree of risk for adverse events to prevent worsening of OA [12]. However, perhaps due to growing awareness of the “opioid epidemic,” concern about possible addiction is becoming one of the key drivers of patient preferences [13]. In a recent US study of OA patient preferences, control of OA pain and symptoms and reduced treatment-related risk of physical dependency were the two most important attributes of a prospective new medicine for adult patients with moderate to severe OA and inadequate response to pain treatment [14].

Prominent health technology assessment organizations such as the public National Institute for Health and Care Excellence in the UK and the private Institute for Clinical and Economic Review in the US recommend the EQ-5D as the preferred measure for HRQoL effects in economic evaluation [15, 16]. In this analysis, we aim to evaluate the relationship between self-reported concerns about becoming addicted to a medicine and individual patient health-related quality of life (HRQoL) measured by (a) the EQ-5D-5L index value and (b) the EQ Visual Analogue Scale (EQ VAS) in patients with OA.

## Methods

This real-world study used patient-level cross-sectional survey data collected between February 01, 2017 and May 31, 2017 from the US Adelphi Disease Specific Programme (DSP)^™^. The Adelphi DSP is a large, multinational platform designed to gather descriptive real-world data on the management of chronic diseases in routine clinical practice, based on physician and patient perspectives [17]. The Adelphi DSP methodology was granted exceptions from requiring ethics approval centrally by the Western Institutional Review Board as it was considered to pose minimal risk to patients and physicians.

Selected physicians (practicing in primary care, rheumatology, or orthopedic surgery and making treatment decisions for at least 10 patients with OA in a typical month) were identified from publicly available lists of healthcare professionals and asked to enroll up to nine consecutive patients and complete corresponding electronic patient record forms with de-identified data. Patients were eligible for inclusion if they had a confirmed diagnosis of OA, were aged 18 years or older, and had provided written informed consent. Patients were not required to be taking a prescription opioid. These participants then completed a patient self-completion survey relevant to OA, in which the patients could respond on a Likert scale of 1–5 to several questions.

For the purposes of this analysis, the question of interest was the item termed “Concern about addiction” (CAA) that was assessed by the patient’s response to the question “I am concerned about becoming addicted to my medicine.” The patient’s response to this question could range from 1 (“completely disagree”) to 5 (“completely agree”). Patients were also required to complete the EQ-5D-5L, a generic, patient-reported measure of health status [18]. The EQ-5D-5L instrument comprises (a) a short descriptive system questionnaire with five dimensions (mobility, self-care, usual activities, pain/discomfort, and anxiety/depression), with five levels of impairment responses, and (b) a health state VAS (0 = worst imaginable health state, 100 = best imaginable health state). Patient responses were linked to a “value set” from the general US population on the five dimensions to generate a utility index value that represents an individual’s health state with anchors at 0 (a state as bad as being dead) to 1 (full health). This index also allows for negative utility values, which theoretically correspond to health states worse than death based on population-assigned weights [19]: states worse than death in patients with OA have been previously reported to be associated with high disability, greater pain severity, and mental distress, as well as some clinical measures such as swollen joint counts [20]. The EQ VAS provides an alternative way for an individual to rate their overall current health.

### Statistical analyses

A set of ordinary least squares (OLS) regressions using HRQoL measures (EQ-5D-5L index value and EQ VAS) as outcomes and CAA as a continuous predictor were developed to estimate the relationship between these measures [21]. The relationship between EQ-5D-5L index value as a predictor and EQ VAS as the outcome was also studied. Finally, with consideration of the EQ VAS as an alternative, patient-specific, and perhaps more general indicator of HRQoL, an OLS regression with the EQ VAS as an outcome and with the EQ-5D-5L index value and the CAA as two independent continuous predictors was estimated in this sample. As a sensitivity analysis, the relationship between CAA and EQ-5D-5L index/EQ VAS was also assessed using a model with CAA as a categorical predictor to explore the linearity assumption.

We used standardized effect size (ES) to gauge the magnitude of effects with 0.2 standard deviation (SD) units considered “small,” 0.5 “medium,” and 0.8 “large” [22]. ESs were calculated as the difference of means of the outcome scores (EQ-5D-5L index value or EQ VAS) from the regression model corresponding to a one category difference on CAA and also as the difference between lowest and highest CAA category, divided by the SD of the corresponding outcome variable.

## Results

A total of 866 patients completed the survey with the majority being female (*n* = 530, 61.2%), Caucasian/White (*n* = 673, 77.7%), and with a mean age of 64.2 years (SD: 11.7). The patient responses to the survey question “I am concerned about becoming addicted to my medicine” were well distributed across categories. Of the 775 who provided a response to this survey question, almost half either disagreed (20.3%) or completely disagreed (27.5%); however, almost three in ten patients either agreed (18.2%) or completely agreed (10.7%) with the statement (the remaining patients neither disagreed or agreed [23.4%]).

**Fig. 1:**
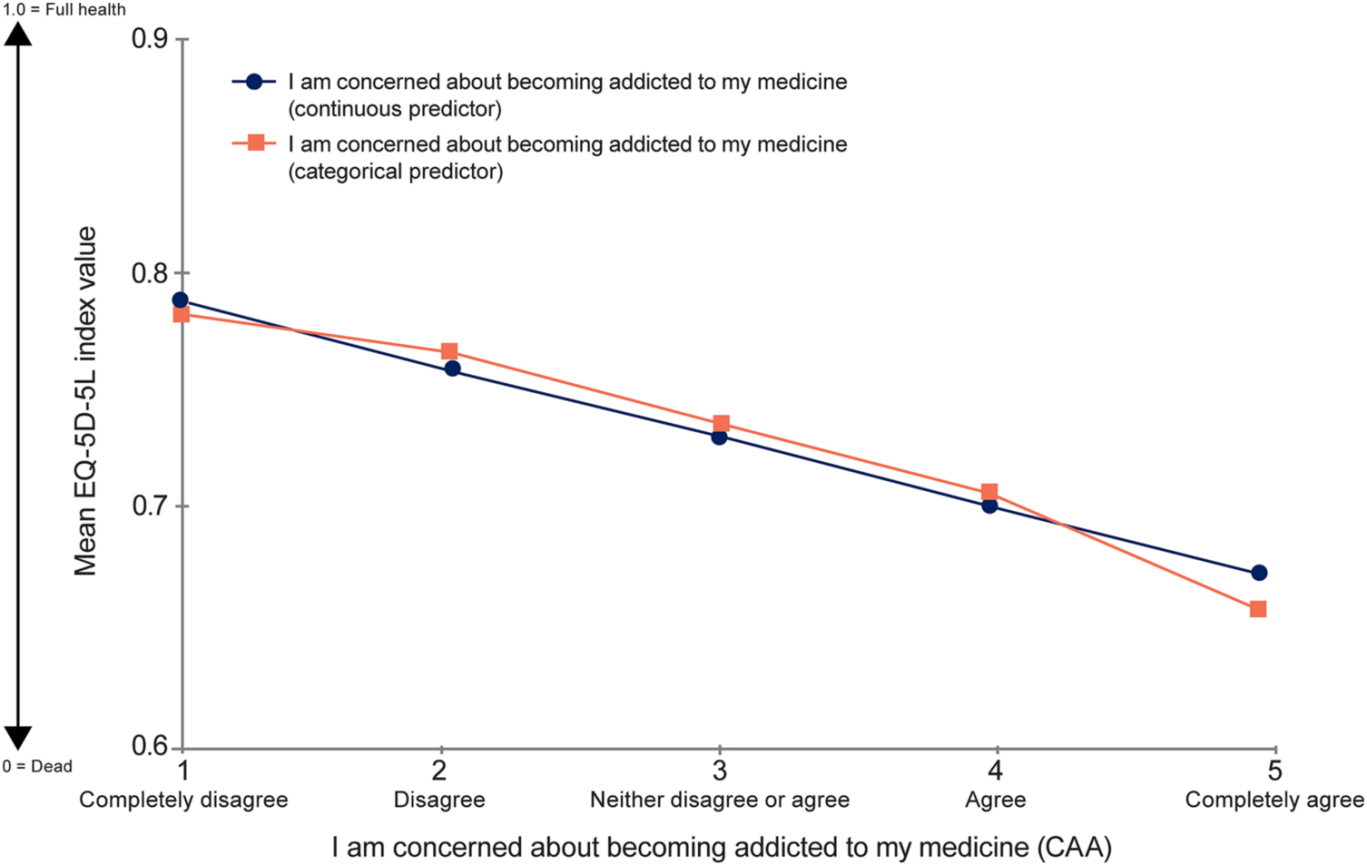
Relationship between EQ-5D-5L index value and CAA score. CAA, “concern about addiction” survey item

When assessing the relationship between CAA and EQ-5D-5L index value using CAA as a continuous predictor variable, OLS regression demonstrated a significant relationship (*n* = 762; R-squared: 0.0359; intercept: 0.82; slope: − 0.029; *p* < 0.0001 for both) between variables (Fig. 1). Each category increase in the CAA response was associated with a reduction of 0.029 in EQ-5D-5L index value, equivalent to a standardized ES of 0.14, which can be interpreted as a “trivial-to-small” effect. The difference in means of 0.11 (*p* < 0.0001) in the EQ-5D-5L index value linked to the difference between the lowest (“Completely disagree”) and the highest (“Completely agree”) CAA category corresponds to the ES of 0.57 (considered a medium effect). Using CAA as a categorical predictor indicated that a linear approximation is appropriate (Fig. 1). A significant correlation of 0.19 (*p* < 0.0001) was observed between CAA and EQ-5D-5L index value.

**Fig. 2:**
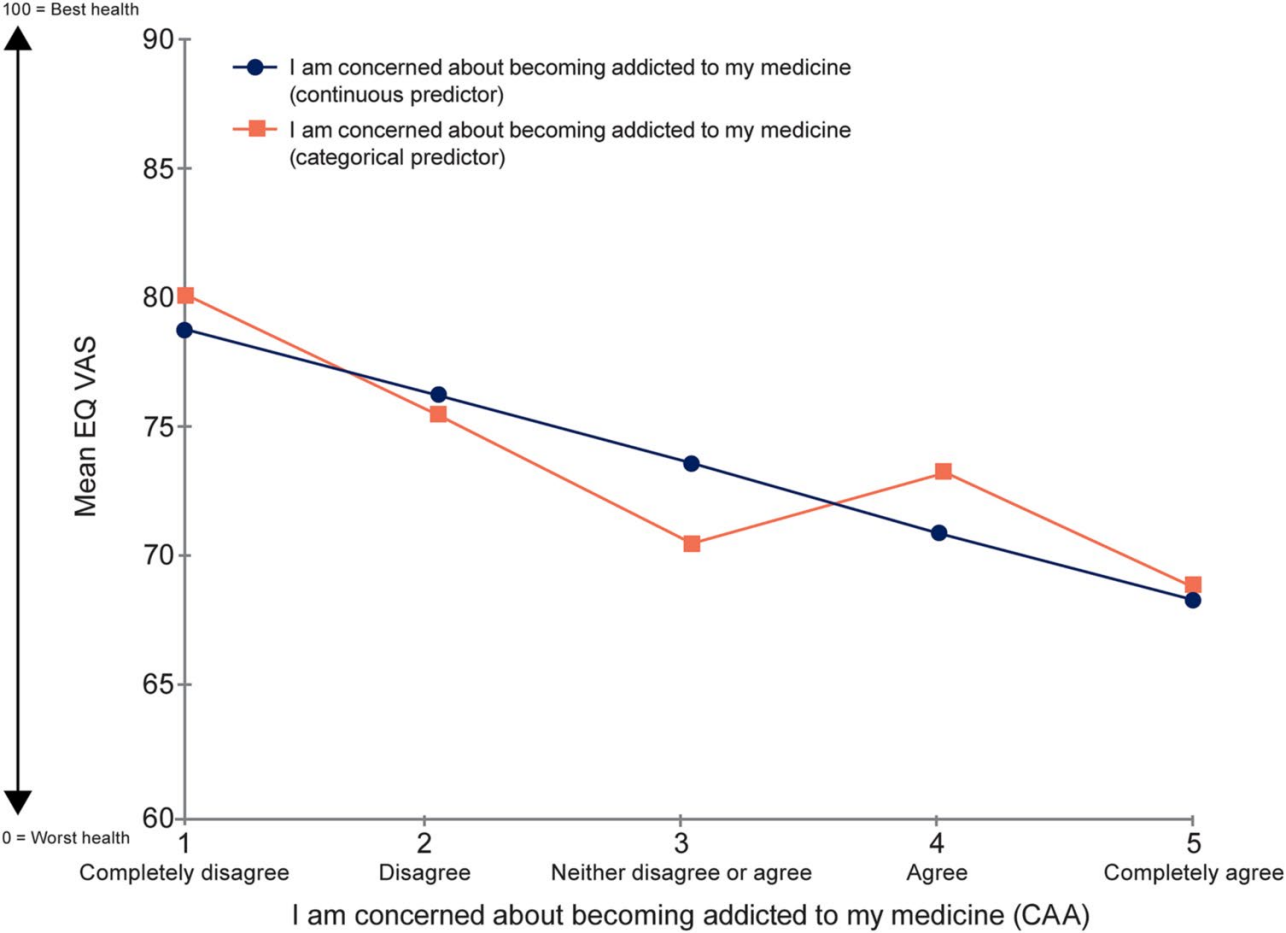
Relationship between EQ VAS and CAA score. CAA, “concern about addiction” survey item; VAS, visual analogue scale

When assessing the relationship between CAA and EQ VAS using CAA as a continuous predictor variable, OLS regression demonstrated a significant relationship (*n* = 761; R-squared: 0.0392; intercept: 81.3; slope: − 2.6; *p* < 0.0001 for both) between variables (Fig. 2). Each category increase in CAA response category was associated with a reduction of 2.6 points in EQ VAS (ES: 0.15). The difference in EQ VAS means linked to the difference between lowest and highest CAA category was 10.5 (*p* < 0.0001), representing an ES of 0.59. Using CAA as a categorical predictor indicated that a linear approximation is appropriate (Fig. 2). A significant correlation of 0.20 (*p* < 0.0001) was observed between CAA and EQ VAS (Fig. 2).

**Fig. 3:**
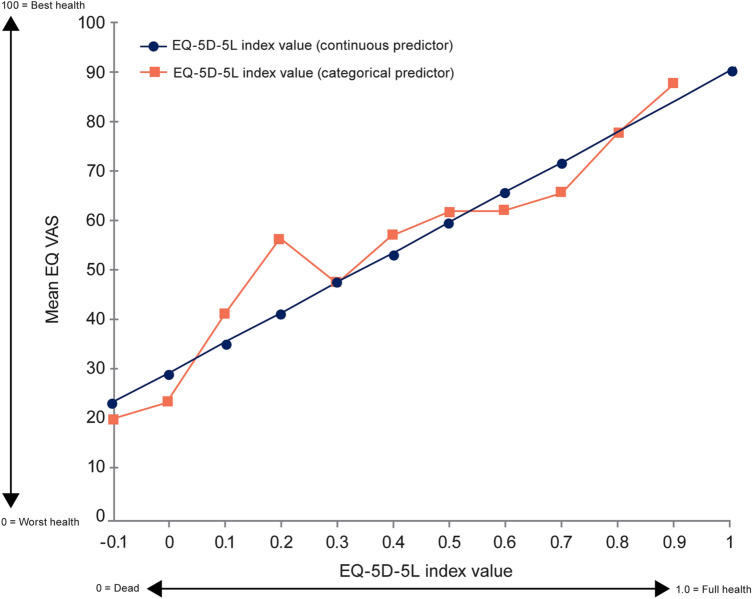
Relationship between EQ VAS vs EQ-5D-5L index value. VAS, visual analogue scale

A significant and robust relationship between EQ VAS as an outcome and EQ-5D-5L index value as a predictor was observed (*n* = 835; R-squared: 0.4695; intercept: 29.1; slope: 60.7; *p* < 0.0001 for both), with a significant correlation between the two measures (0.69; *p* < 0.0001) (Fig. 3). Using EQ-5D-5L index value as a categorical predictor indicated that a linear approximation is appropriate.

When EQ-5D-5L index value and CAA scores were used simultaneously as predictors of EQ VAS, the effect of CAA (after adjusting for EQ-5D-5L index) remained significant (*n* = 754; R-squared: 0.4676; slope: −  0.97; *p* = 0.0071) (Table 1). In this case, the difference in EQ VAS means corresponding to the difference between lowest and highest CAA category was 3.89, with an associated ES of 0.22, which would be regarded as “small.” This is, however, equivalent to − 0.039 on a utility scale of 0–1.0 (and that allows for negative utility values, which theoretically correspond to health states worse than death based on population-assigned weights [19]), which would be regarded as significant in utility and economic terms. Lastly, after adjustment in this model for age, gender, and ethnicity, the effect of CAA was still statistically significant (*p* = 0.0129) and very similar in magnitude (slope: − 0.92).

**Table 1:** Predicting EQ VAS with EQ-5D-5L index value and CAA

Effect	Estimate	Standard error	*p* value
Intercept	33.56	2.20	< 0.0001
EQ-5D-5L	58.41	2.38	< 0.0001
CAA	− 0.97	0.36	0.0071

*CAA* “concern about addiction” survey item

*VAS* visual analogue scale

## Discussion

This analysis used patient-completed questionnaire data consisting of a Likert survey question about CAA and one of the most well-established generic HRQoL measures, the EQ-5D-5L. In doing so, we found that patients with a diagnosis of OA who have concerns about medication addiction (as indicated by self-reported CAA) have significantly different EQ-5D-5L index and EQ VAS scores compared with patients who do not express such concerns. Furthermore, when EQ-5D-5L index and CAA measures were used simultaneously to predict EQ VAS, CAA had a small, incremental predictive effect beyond that observed for EQ-5D-5L index. This suggests that CAA may have an additional negative impact, which might not be reflected in EQ-5D-5L index values. Such a finding could be of clinical and economic importance.

Based on these results, it could be hypothesized that an alternative, effective, non-opioid, or otherwise non-addictive, pain relief therapy would be accompanied by improved CAA Likert scores of patients receiving or considering such a therapy (i.e. concern about addiction and consequent CAA scores would presumably be alleviated by availability of an equally efficacious non-addictive therapy): this would imply improved patient HRQoL and a utility gain, resulting in greater quality-adjusted life years gained in an economic “cost-utility analysis.” Based on this, it may be reasonable to assume that health technology assessment authorities who currently rely on the EQ-5D-5L may underestimate the value of products that reduce concerns about addiction/dependency. One potential solution for this would be to introduce a “bolt-on” question to the EQ-5D-5L for patients with OA, specifically aimed (after appropriate psychometric validation) at addressing these concerns in assessments of the impact of new interventions in these patients [23]. EQ-5D has been criticized for being insensitive or failing to capture important aspects of health for some conditions [24, 25]. One possible solution on how best to obtain health state preference data is the development of new dimensions to “bolt-on” to existing generic preference-based measures. The development of these bolt-on item(s) to the EQ-5D could enable researchers to retain the EQ-5D descriptive system as core and select additional dimensions to improve the content validity of the instrument for a particular condition. Several studies have investigated the inclusion of additional dimensions to the EQ-5D, including a cognitive dimension [26] and a sleep dimension [27], demonstrating a significant impact on health state values of EQ-5D in the case of the cognitive dimension study. Alternatively, given the distribution of CAA in a target population, the estimates here could be used to approximate the value of a shift to no concern about addiction.

To our knowledge, this study is the first to use rigorous methodologies to estimate the “disutility” (i.e. negative impact on patient HRQoL) of concern about medication addiction in OA. Of note, several different measurement instruments are available in weighing the impact of CAA on patient HRQoL. One of the most widely used disease-specific measures of OA symptoms is the Western Ontario and McMaster Universities Osteoarthritis Index (WOMAC)^©^.^1^ While the WOMAC is commonly used in clinical studies, it is not suitable for direct use in conventional economic evaluation because WOMAC scores provide neither a cardinal nor a preference-based index scale. Therefore, economic evaluations sometimes rely on mapping from WOMAC to predict EQ-5D-5L and studies have shown consistent statistical relationships between the two with demonstrated goodness of fit [28]. It would be worthwhile to explore the robustness of this CAA effect with other measurement instruments.

There is growing awareness of the importance of including the patient perspective when assessing clinical outcomes and informing drug development decisions [29]. In this study, nearly one-third of patients with OA were concerned about addiction to medication. Although the concern may not be limited to opioids, patients being treated for chronic pain have reported drug addiction associated with opioids as one of their main fears [30]. Likewise, primary care physicians report concerns of drug addiction when prescribing opioids for chronic pain [31]. These concerns, along with the potential negative impact on patient HRQoL, highlight the importance of developing new efficacious and safe medications without addictive properties for the treatment of OA.

This exploratory analysis has certain limitations. Patients were drawn from a small number of US physicians and specialties, and may not be representative of all physicians in the US who treat OA. In turn, this may have resulted in patient selection bias, although the selection of consecutive patients was required to reduce this bias. Key data related to patient concern over medication addiction was based on a response from one Likert survey question. Furthermore, patient concerns about addiction or dependence may extend beyond opioids to other agents used to treat pain, such as benzodiazepines or antidepressants. In the case of antidepressants, the management of withdrawal symptoms upon discontinuation can be difficult [32, 33]. Lastly, although a significant relationship was demonstrated between CAA and HRQoL, this does not confirm causality or specifically that HRQoL genuinely differs *as a result* of the level of one’s CAA. For example, individuals with poorer HRQoL may be more likely to express CAA. In such cases, it is not necessarily that CAA impacts HRQoL per se, but that poorer HRQoL is linked to greater CAA.

In conclusion, our findings suggest that concern about medication addiction in patients with OA may have an impact on patient HRQoL, with patients more concerned with addiction possibilities reporting poorer HRQoL outcomes. Additionally, it may be the case that some aspects of CAA in these patients are not reflected by the standard EQ-5D-5L index, which is often used in economic evaluations presented to health technology assessment authorities.

## Data Availability

Upon request, and subject to certain criteria, conditions, and exceptions (see https://www.pfizer.com/science/clinical-trials/trial-data-and-results for more information), Pfizer will provide access to individual de-identified participant data from Pfizer-sponsored global interventional clinical studies conducted for medicines, vaccines, and medical devices (1) for indications that have been approved in the US and/or EU or (2) in programs that have been terminated (i.e. development for all indications has been discontinued). Pfizer will also consider requests for the protocol, data dictionary, and statistical analysis plan. Data may be requested from Pfizer trials 24 months after study completion. The de-identified participant data will be made available to researchers whose proposals meet the research criteria and other conditions, and for which an exception does not apply, via a secure portal. To gain access, data requestors must enter into a data access agreement with Pfizer.

## Footnotes

1 © 1996 Nicholas Bellamy. WOMAC^®^ is a registered trademark of Nicholas Bellamy (CDN, EU, USA).
